# Protocol to quantitatively assess glycolysis and related carbon metabolic fluxes using stable isotope tracing in Crabtree-positive yeasts

**DOI:** 10.1016/j.xpro.2025.103786

**Published:** 2025-04-22

**Authors:** Shreyas Niphadkar, Sreesa Sreedharan, Vineeth Vengayil, Sunil Laxman

**Affiliations:** 1 Institute for Stem Cell Science and Regenerative Medicine (BRIC inStem), GKVK Post Bellary Road, Bangalore 560065, India; 2 School of Chemical and Biotechnology, SASTRA Deemed to be University, Thanjavur 613401, India

**Keywords:** metabolism, metabolomics, model organisms, systems biology

## Abstract

Crabtree-positive yeasts rapidly consume glucose via glycolysis, making it difficult to experimentally estimate their actual glycolytic rate or flux. We present a stable isotope labeling and liquid chromatography-tandem mass spectrometry (LC-MS/MS)-based protocol to quantitatively estimate glycolytic and related carbon metabolic fluxes using *Saccharomyces cerevisiae*. This approach defines time windows to capture glucose metabolic intermediate production before label saturation, enabling a comparison of glycolytic flux changes across different cells. This protocol provides a reliable, quantitative approach to study dynamic metabolic fluxes in these cells.

For complete details on the use and execution of this protocol, please refer to Vengayil et al., 2024.^1^

## Highlights

• ^13^C tracing for glycolysis, PPP, and TCA fluxes in Crabtree-positive yeasts
• Defining time windows to track flux changes in pathways with zero-order kinetics
• Detailed procedure for rapid pulsing and quenching to quantify glycolysis fluxes

## Abstract (editor highlights)

Publisher’s note: Undertaking any experimental protocol requires adherence to local institutional guidelines for laboratory safety and ethics.

## Graphical abstract

**Figure:**
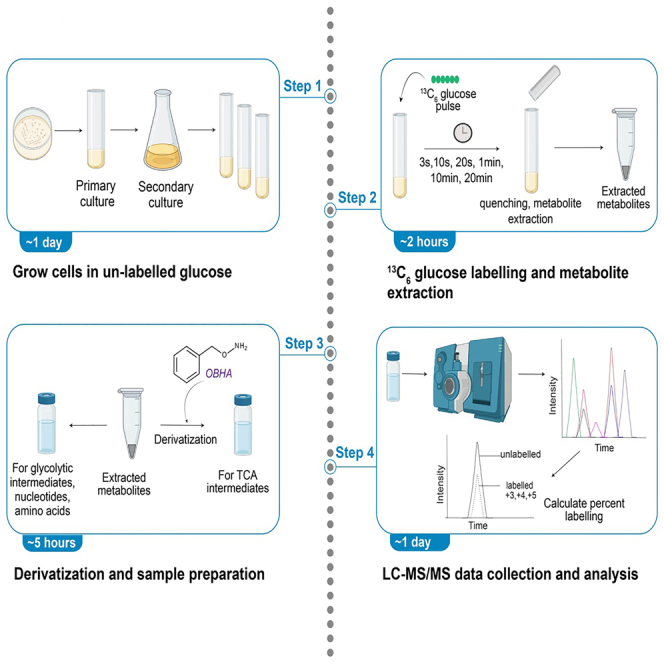

## Before you begin

**Figure 1:**
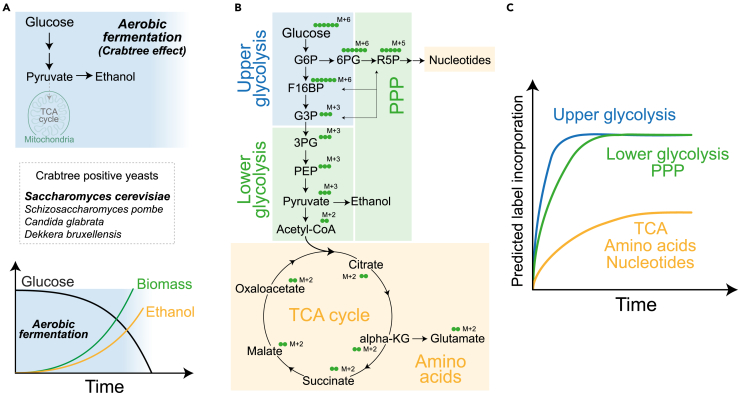
Labeling kinetics of glucose metabolic pathways in Crabtree positive cells (A) Schematic describing aerobic fermentation in Crabtree positive yeasts. (B) Schematic describing ^13^C label incorporation from ^13^C-glucose into central carbon metabolic pathways, indicating expected mass changes coming from labeled carbon incorporation. (C) Predicted ^13^C labeling kinetics in central carbon metabolic pathways following a pulse of ^13^C-glucose. The time axis at this stage is arbitrary, but the trends of label saturation are indicated.

Crabtree positive yeasts (widely used in research and industrial applications) rapidly convert sugars to ethanol through fermentation, regardless of the presence of oxygen^2^ (Figure 1A). These include large clades of important yeasts in the food, beverage, biotechnology industries, basic research, and pathogens, including - *Saccharomyces cerevisiae*, *S. pastorianus*, *S. eubayanus*, *Candida glabrata*, *Schizosaccharomyces pombe* and several other species. Despite the presumed inefficiency of incomplete glucose oxidation, high rates of ‘wasteful’ glycolysis can sufficiently support energetic and biosynthetic needs.^3^^,^^4^^,^^5^ However, current analytical methods struggle to accurately measure these rapid glycolytic rates, because these cells operate at near saturation of glycolytic rates.^1^^,^^6^

Current methods for approximating glycolytic rates include measuring ethanol secretion, performing enzymatic assays for glycolytic enzyme activities, using fluorescence sensors to quantify glycolytic metabolite levels, and estimating the abundances of glycolytic enzymes, or use indirect indicators like changes in extracellular pH due to the secretion of lactic acid.^7^^,^^8^^,^^9^ While these approaches provide an approximation of overall glycolytic flux, they make several assumptions, and cannot reveal any regulatory aspects of specific steps or nodes within glycolysis and associated glucose metabolic pathways.

In contrast to these approaches, stable isotope tracing using ^13^C -labeled glucose offers the potential to dissect rates and regulation at individual steps within glycolysis.^10^^,^^11^ However, most studies using ^13^C- glucose are in cells where the rates of glycolysis etc. are considerably slower - by an order of magnitude or more. In these cases, given the slower rates, the label does not saturate and glycolytic rates can be measured and compared across cell types. These measurements however becomes more challenging when applying this technique to pathways or systems operating at near saturation rates, or at zero-order kinetics, like glycolysis in Crabtree positive cells.^12^ Here, any stable-isotope label incorporation into glycolytic intermediates saturates very rapidly (reaching a ‘steady state’), and steady-state estimates of unlabeled intermediates cannot distinguish between production-consumption of metabolites, all of which makes it difficult to track changes in glycolytic and related metabolic fluxes. Therefore, sensitive, quantitative methods to assess glycolytic flux, by defining precise time windows before label-saturation, as well as assessing differences in kinetics of saturation of different arms of glucose metabolism are critical for such cells (Figures 1B and 1C).

**Figure 2:**
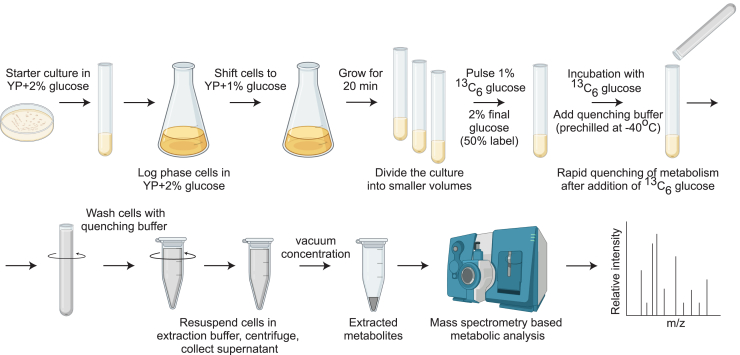
A detailed workflow to follow in order to correctly quantify glycolysis and related carbon metabolic fluxes using ^13^C glucose pulse and stable isotope tracing

In a recent report, we found that a pulse of ^13^C glucose saturates into glycolytic intermediates within 10 s.^1^ Therefore, any measurement beyond this time point will not give actual changes in flux. This is precisely what we would expect for pathways operating at zero order kinetics i.e., the upper part of glycolysis will saturate very fast, followed by the pentose phosphate pathway (PPP) and the lower part of glycolysis and other distant outputs.^12^ In this detailed method, we clearly define and establish optimal time windows for quantitatively estimating or comparing glycolytic rates using ^13^C-glucose, where we could see linear increase in label incorporation into intermediates without attaining saturation (Figure 2). We extend this analysis to calculate time windows for measuring flux through related pathways, including the pentose phosphate pathway (PPP), tricarboxylic acid (TCA) cycle, and amino acid and nucleotide synthesis. This approach now enables comparisons of glycolytic and related metabolic fluxes between cells with differing glycolytic rates, and allows quantitative estimates of glucose usage and diversion to different arms of carbon metabolism in Crabtree positive yeasts. This detailed, quantitative method will enable users to quantitatively assess glucose metabolism in such cells, which is of high importance in basic research, and for metabolic engineering applications widely used in industry.

### Preparation for yeast growth

**Timing: ∼2 h**

1. Prepare and autoclave yeast growth media.
  ***Note:*** YP media used in this protocol is prepared as described in the materials and equipment section for a required volume. For solid media, agar is added.
  a. Weigh the required amounts of yeast extract, peptone according to desired final volume.
  b. Dissolve the weighed components in distilled water.
  c. Autoclave the solution at 121°C for 30 min.
  d. Once cooled, add filter-sterilized glucose to a final concentration of 2% to the media
    ***Note:*** YP with 1% or 2% glucose is used in this protocol as described in the Step-by-step method details section of growing cells. Unless stated otherwise, use YP with 2% glucose throughout the protocol. For shifting cells prior to label addition, use YP with 1% glucose as specified in the Step-by-step method details section of growing cells.
2. Aliquot 16 mL of YP (2% glucose) into labeled conical flasks for starting cultures as mentioned in Step-by-step method details section of growing cells.
3. Label 50 mL conical tubes and 1.5 mL vials for shifting the cells as specified in Step-by-step method details section of growing cells.

### Preparation for metabolite extraction

**Timing: ∼40 min**

4. Prepare quenching (Buffer Q) and extraction (Buffer E) buffers in required volumes as described in the materials and equipment section.
  a. Quenching buffer (Buffer Q): 60% methanol (v/v). Prepare ∼1000 mL of quenching buffer by mixing methanol with distilled water.
    ***Note:*** The solution can be prepared and stored at −20°C.
  b. Extraction buffer (Buffer E): 75% ethanol (v/v). Prepare ∼40 mL of extraction buffer by mixing the required volume of ethanol (MS grade) with water (LC-MS grade).
    ***Note:*** This buffer is prepared fresh before the extraction and stored at room temperature.
5. Label the required number of 50 mL conical tubes and 2 mL microcentrifuge vials for the extraction.
6. Aliquot 40 mL of chilled quenching buffer into the conical tubes.

***Note:*** Pre-chill these conical tubes at −40°C before the extraction. For this, keep the conical tubes containing quenching buffer in a dewar containing 60% methanol. Maintain the temperature of the dewar at −40°C by adding dry ice.

**CRITICAL:** Monitor the temperature of the dewar and maintain it between −40°C to −45°C using dry ice. Do not cool below −50°C as cells will freeze and ice over at that temperature.

7. Maintain centrifuges at −5°C.
8. Maintain a heating block at 80°C.
9. Prepare 50% ^13^C_6_ glucose solution.
  a. Weigh the required amount (2 g for 4 mL solution) of ^13^C_6_ glucose.
  b. Dissolve it in distilled water to make a 50% solution (∼4 mL of 50% ^13^C_6_ glucose can be prepared).

***Note:*** This solution is prepared fresh before extraction. Store this solution at room temperature.

## Key resources table

**Table:** 

REAGENT or RESOURCE	SOURCE	IDENTIFIER
**Experimental models: Organisms/strains**		

*Saccharomyces cerevisiae*: CEN.PK: Mat “a”	van Dijken et al.^13^	
*Saccharomyces cerevisiae*: CEN.PK: Mat “a”; *tdh2Δtdh3Δ*::G418+NAT	Vengayil et al.^1^	

**Chemicals, peptides, and recombinant proteins**		

Yeast extract	Gibco	Cat #212750
Peptone	Gibco	Cat #211677
D-glucose	Qualigens	Cat #50-99.5
Agar	Gibco	Cat #214010
^13^C_6_ glucose	Cambridge Isotope Laboratories	Cat #110187-42-3
Methanol	Qualigens	Cat #67-56-1
Methanol (LC-MS grade)	Fischer Scientific	Cat #A456-4
Ethanol (LC-MS grade)	Merck	Cat #1.00983
Water (LC-MS grade)	Fischer Scientific	Cat #W6-4
Acetonitrile (LC-MS grade)	Fischer Scientific	Cat #A955-4
1-ethyl-3-(3-dimethylaminopropyl) carbodiimide (EDC)	Sigma-Aldrich	Cat #03449
O-benzylhydroxylamine (OBHA)	Sigma-Aldrich	Cat #B22984
Pyridine	Sigma-Aldrich	Cat# 270970
Ethylacetate	Sigma-Aldrich	Cat #270989
Formic acid	Merck	Cat #5330020050
Ammonium acetate	Sigma-Aldrich	Cat# 431311
HCl	Qualigens	Cat# 7646-01-0

**Software and algorithms**		

Analyst 1.6.2 software (Sciex)	Sciex	N/A
MultiQuant version 3.0.1 (Sciex)	Sciex	N/A

**Other**		

Autosampler vials	Thermo Scientific	Cat #03-340-620
Dual position snap cap tubes	SPL Life Sciences	Cat #40014
1.5 mL microcentrifuge tubes	Tarsons	Cat# 500010
2 mL microcentrifuge tubes	Tarsons	Cat# 500020
50 mL conical tubes	Tarsons	Cat# 546041
Synergi 4 μm Fusion-RP 80Å (150 × 4.6 mm)	Phenomenex	Cat# 00F-4424-E0
Nexera UHPLC	Shimadzu	N/A
5500 QTRAP mass spectrometer	AB SCIEX	N/A

## Materials and equipment

**Table:** Media composition

Components	Final concentration	Amount
Yeast Extract	1% (w/v)	5 g
Peptone	2% (w/v)	10 g
Agar	2% (w/v)	10 g
Glucose (50%)	2% (w/v)	20 mL
Distilled water	N/A	480 mL
**Total**	**N/A**	**500 mL**

***Note:*** A 50% glucose stock solution can be prepared and filter sterilized for media preparations. This can be stored aseptically at room temperature.

### LC and mass spectrometer

Synergi 4 μm Fusion-RP 80Å (150 × 4.6 mm, Phenomenex) LC column is used for separation of metabolites. Shimadzu Nexera UHPLC system with a triple quadrupole 5500 QTRAP mass spectrometer (AB SCIEX) is used in this protocol for the analysis. .The flow and mass spectrometric parameters are adapted from^12^ and described in detail in this protocol in Tables 1, 2, 3, and 4.

**Table 1:** Composition of mobile phase solvents for HPLC-based separation

Mobile phase solvents for positive polarity MS mode:	
Components	Final concentrations
**Solvent A**	

Water	99.90%
Formic acid	0.10%

**Solvent B**	

Methanol	99.90%
Formic acid	0.10%

Mobile phase solvents for negative polarity MS mode:	
Components	Final concentration
**Solvent A′**	

Water	
Ammonium acetate	5 mM

**Solvent B′**	

Acetonitrile	100%

Note: Use LC-MS grade reagents to make these solvents

**Table 2:** HPLC gradient parameters for separating metabolites

LC gradient parameters for positive polarity mode	
Column	Synergi 4 μm Fusion-RP 80Å (150 × 4.6 mm, Phenomenex)
Column temperature	40°C
Injection volume	10 μL
Flow rate	0.4 mL/min

Time	Gradient B%
0	0
3	5
10	60
11	80
12	80
12.1	0
17	0
17.1	stop

LC gradient parameters for positive polarity mode for derivatized samples	
Time	Gradient B%
0	50
2	75
6	100
15	100
17	50
21	50
21.1	stop

LC gradient parameters for negative polarity mode	
Column	Synergi 4 μm Fusion-RP 80Å (150 × 4.6 mm, Phenomenex)
Column temperature	25°C
Injection volume	10 μL
Flow rate	0.4 mL/min

Time	Gradient B%
0	0
3	5
10	60
11	95
14	95
15	5
16	0
21	stop

**Table 3:** MS setting

**Parameters**	

Scan type	MRM
Ion source	Electrospray ionization

**Source parameters**	

Curtain gas	30 psi
Collision gas	Medium
Ion spray voltage	±4500 eV
Temperature	400°C
Ion Source Gas 1	30 psi
Ion Source Gas 2	30 psi
Declustering potential	±65 eV
Entrance potential	±11.4 eV
Collision cell exit potential	±12 eV

**Table 4:** Metabolite specific MRM settings

Metabolite	Parent ion mass	Fragment ion mass	CE (eV)
**MS positive polarity MRM settings**			

Glutamine	147	130	6
Gln_13C_1	148	131	6
Gln_13C_2	149	132	6
Gln_13C_3	150	133	6
Gln_13C_4	151	134	6
Gln_13C_5	152	135	6
Glutamate	148	130	7
Glu_13C_1	149	131	7
Glu_13C_2	150	132	7
Glu_13C_3	151	133	7
Glu_13C_4	152	134	7
Glu_13C_5	153	135	7
Aspartate	134	74	15
Asp_13C_1	135	74	15
Asp_13C_2	136	74	15
Asp_13C_3	137	75	15
Asp_13C_4	138	76	15
Alanine	90	44	11
Ala_13C_1	91	45	11
Ala_13C_2	92	46	11
Ala_13C_3	93	46	11
AMP	348	136	21
AMP_13C_5	353.2	136	21
Serine	106	60	13
Ser_13C_1	107	61	13
Ser_13C_2	108	62	13
Ser_13C_3	109	62	13
GMP	364	152	18
GMP_13C_5	369.2	152	18

**MS positive polarity MRM settings for derivatized samples**			

Pyruvate	299	181	15
Pyruvate_13C_1	300	181	15
Pyruvate_13C_2	301	181	15
Pyruvate_13C_3	302	181	15
Citrate	508	91.2	25
Citrate_13C_1	509	91.2	25
Citrate_13C_2	510	91.2	25
Citrate_13C_3	511	91.2	25
Citrate_13C_4	512	91.2	25
Citrate_13C_5	513	91.2	25
Citrate_13C_6	514	91.2	25
2-KG	462	91.2	25
2KG_13C_1	463	91.2	25
2KG_13C_2	464	91.2	25
2KG_13C_3	465	91.2	25
2KG_13C_4	466	91.2	25
2KG_13C_5	467	91.2	25
Succinate	329	206	15
Succinate_13C_1	330	207	15
Succinate_13C_2	331	208	15
Succinate_13C_3	332	209	15
Succinate_13C_4	333	210	15
Malate	345	91.2	33
Malate_13C_1	346	91.2	33
Malate_13C_2	347	91.2	33
Malate_13C_3	348	91.2	33
Malate_13C_4	349	91.2	33

**MS negative polarity MRM settings**			

G6P	259.02	97	−20
G6P_13C_1	260.02	97	−20
G6P_13C_2	261.02	97	−20
G6P_13C_3	262.02	97	−20
G6P_13C_4	263.02	97	−20
G6P_13C_5	264.02	97	−20
G6P_13C_6	265.02	97	−20
F16BP	339	97	−20
F16BP_13C_6	345	97	−20
G3P	169	97	−20
G3P_13C_1	170	97	−20
G3P_13C_2	171	97	−20
G3P_13C_3	172	97	−20
3PG	185	97	−20
3PG_13C_1	186	97	−20
3PG_13C_2	187	97	−20
3PG_13C_3	188	97	−20
PEP	167	79	−12
PEP_13C_1	168	79	−12
PEP_13C_2	169	79	−12
PEP_13C_3	170	79	−12
6PG	275.02	97	−20
6PG_13C_1	276.02	97	−20
6PG_13C_2	277.02	97	−20
6PG_13C_3	278.02	97	−20
6PG_13C_4	279.02	97	−20
6PG_13C_5	280.02	97	−20
6PG_13C_6	281.02	97	−20
R5P	229.01	97	−20
R5P_13C_1	230.01	97	−20
R5P_13C_2	231.01	97	−20
R5P_13C_3	232.01	97	−20
R5P_13C_4	233.01	97	−20
R5P_13C_5	234.01	97	−20
S7P	289.03	97	−20
S7P_13C_1	290.03	97	−20
S7P_13C_2	291.03	97	−20
S7P_13C_3	292.03	97	−20
S7P_13C_4	293.03	97	−20
S7P_13C_5	294.03	97	−20
S7P_13C_6	295.03	97	−20
S7P_13C_7	296.03	97	−20
UDP-Glc	565	323	−25
UDP-Glc_13C_1	566	323	−25
UDP-Glc_13C_2	567	323	−25
UDP-Glc_13C_3	568	323	−25
UDP-Glc_13C_4	569	323	−25
UDP-Glc_13C_5	570	323	−25
UDP-Glc_13C_6	571	323	−25

Complete names of metabolites are now added in the table legends.

Parent and fragment ion masses and the collision energy for each of the metabolites are provided. AMP, adenosine monophosphate; GMP, guanosine monophosphate; 2-KG, 2-ketoglutarate; G6P, glucose-6-phosphate; F16BP, fructose-1,6-bisphosphate; G3P, glyceraldehyde-3-phosphate; 3PG, 3-phosphoglycerate; PEP, phosphoenolpyruvate; 6PG, 6-phosphogluconate; R5P, ribose-5-phosphate; S7P, sedoheptulose-7-phosphate; UDP-Glc, uridine diphosphate glucose.

### Buffers/solutions

• ^13^C_6_ glucose: Make 50% stock solution by dissolving the required amount in water. Vortex to mix and store at room temperature.
• Quenching buffer (Buffer Q): 60% methanol (v/v). Maintain at −20°C before use.
• Extraction buffer (Buffer E): 75% ethanol (v/v). Prepare fresh and store at room temperature.
• Pyridine buffer: For 100 mL, add 8.6 mL of pyridine and 86 mL of water and mix by constant stirring. Adjust the pH to 5.0, using 5.4 mL HCl (12.1 M). Store at room temperature.

**CRITICAL:** Handle HCl and pyridine in a fume hood. Wear chemical resistant gloves and protective goggles to ensure safe handling.

• 1 M EDC in pyridine buffer. Weigh required amount of EDC in a microcentrifuge tube and dissolve in pyridine buffer to make 1 M EDC (∼1 mL of 1 M EDC can be prepared). This solution should be freshly prepared before the derivatization.
• 0.5 M OBHA in pyridine buffer. Weigh required amount of OBHA in a microcentrifuge tube and dissolve in pyridine buffer to make 0.5 M OBHA (∼1 mL of 0.5 M OBHA can be prepared). This solution should be freshly prepared before the derivatization.

## Step-by-step method details

### Growing cells

**Timing: ∼1 day**

This step describes preparation of yeast cells for ^13^C glucose pulse labeling and metabolite extraction.

1. Inoculate a single yeast colony from an agar plate in ∼4 mL of YPD (YP medium with 2% glucose) media in a sterile dual position snap cap tube. Incubate overnight with shaking at 30°C, 240 rpm.

***Note:*** We recommend to include biological replicates and process them together using the same batch of media. For this protocol, we have used 3 biological replicates.

2. Use this primary culture to start a secondary culture in 16 mL of fresh YPD (YP medium with 2% glucose).
  a. Measure the optical density of primary culture.
  b. Dilute it in fresh YPD medium to achieve OD_600_ of 0.2.
3. Grow cells to early log phase (OD600: 0.8-1).

***Note:*** Cell growth can be monitored by measuring the optical density at 600 nm (OD600). Aim for an OD600 between 0.8 and 1.0 which generally corresponds to early log phase. This would usually take 4 h for wild type cells.

4. Harvest cells by decanting the cultures into 50 mL conical tubes and centrifuge at 1000 × *g* for 2 min at room temperature.
5. Discard the supernatant, resuspend the cell pellet in the 15 mL of fresh YP media with 1% glucose and transfer it to a conical flask.
6. Incubate the cultures for another 20 min at 30°C, 240 rpm.
7. Divide the culture into 50 mL conical tubes (depending on the number of time points, see step 8), with each tube containing ∼5 OD cells OD_600_ cells (∼5–6 mL of expected culture volume).

### Sample collection and metabolite extraction

**Timing: ∼2 h**

This step describes ^13^C glucose labeling at different time points and subsequent metabolite extraction.

***Note:*** Pre-chill conical tubes containing buffer Q at −40°C before the extraction. For this, keep the conical tubes with quenching buffer in dewar containing 60% methanol. Maintain the temperature of the dewar at −40°C by adding dry ice.

8. To each of the tubes containing ∼5 OD_600_ log phase cells, add freshly prepared ^13^C_6_ glucose to a final concentration of 1% (∼100 μL for 5 mL of 5 OD_600_ cells).
  ***Note:*** The final glucose concentration would now become 2% with 1% unlabeled glucose and 1% labeled glucose).
  **CRITICAL:** Shake the tubes manually for a particular time and quickly add 40 mL of chilled quenching buffer (Buffer Q) (maintained at −40°C).
  a. For measurement at short time points (such as 3, 10, 30 s after ^13^C_6_ glucose addition) manually shake the tubes and quickly add chilled quenching buffer at these intervals. Use a timer to ensure accuracy during this step.
  b. For measurement at longer time points (1 min or more) keep the tubes back in the shaker after addition of ^13^C_6_ glucose, take out the tubes and quickly add chilled quenching buffer at these intervals.
9. Maintain the tubes in the −40°C dewar for 5 min.
10. Centrifuge the tubes at 1000 × *g* for 3 min at −5°C.
11. Decant the supernatant, add 1 mL chilled quenching buffer and resuspend the cell pellet by pipetting. Transfer the cell suspension to a 2 mL tube.
12. Centrifuge the 2 mL tubes for 1000 × *g* at −5°C for 2 min and decant the supernatant.
13. Add 1 mL of extraction buffer (Buffer E) to the cell pellet, resuspend the cells by vortexing for ∼20 s.
14. Heat the 2 mL tubes for 3 min at 80°C and immediately transfer the tubes to ice bath and incubate for 5 min.
15. Spin the tubes at 20000 × *g* for 1 min at room temperature and transfer 950 μl of supernatant to a fresh 1.5 mL tube.
16. Again centrifuge for 20000 × *g* for 10 min at room temperature and transfer 900 μL of supernatant to a fresh tube.

***Note:*** At this step, the metabolite extracts can be divided into three fractions for detection of metabolites with or without derivatization.

17. Dry the samples using a vacuum concentrator.

**Pause Point:** Samples can be stored at −80°C for a few weeks before LC-MS/MS analysis. However, to ensure the accurate quantitation of certain TCA cycle metabolites that are unstable in aqueous environments, performing OBHA derivatization immediately after extraction ensures their reliable measurement. In particular, aspartate, malate and fumarate from the TCA cycle are not stable over several days even when stored at −80°C.

### Sample preparation for mass spectrometry and derivatization

**Timing: ∼5 h**

This step describes sample preparation for LC-MS/MS analysis and derivatization for TCA metabolites.

***Note:*** Metabolites were measured without derivatization to assess the incorporation of ^13^C carbon into the intermediates of glycolysis, PPP and amino acids.

TCA cycle metabolites containing functional carboxyl groups can be effectively derivatized using O-benzylhydroxylamine (OBHA). We employed a previously optimized protocol for derivatization and LC-MS/MS detection.^12^

18. Dissolve the metabolite extract in 50 μL of LC-MS grade water and add 50 μL of 1 M 1-ethyl-3-(3-dimethylaminopropyl) carbodiimide (EDC) and mix thoroughly by shaking for 10 min at room temperature.
19. Add 100 μL of 0.5 M OBHA and shake on a mixer for 1 h.
20. Add 300 μL of ethylacetate to the reaction mixture and shake for 10 min.
21. Transfer the top layer to a fresh tube. Repeat step 19 two more times and pool the top layers.
22. Dry the derivatized extract using vacuum concentrator.

**Pause Point:** The derivatized samples can be stored at −80°C till further use.

### LC-MS/MS analysis

**Timing: ∼1 day**

This section covers the mass spectrometry based metabolic analysis. The mass spectrometers used in these studies were an AB Sciex 5500 Triple quadrupole, or AB Sciex 6500 QTRAP triple quadrupole systems. This method is easily adaptable to any triple-quadrupole mass spectrometer, as well as other quantitative mass spectrometers with a large linear dynamic range (for example SCIEX 7600 ZenoTOF).

**Figure 3:**
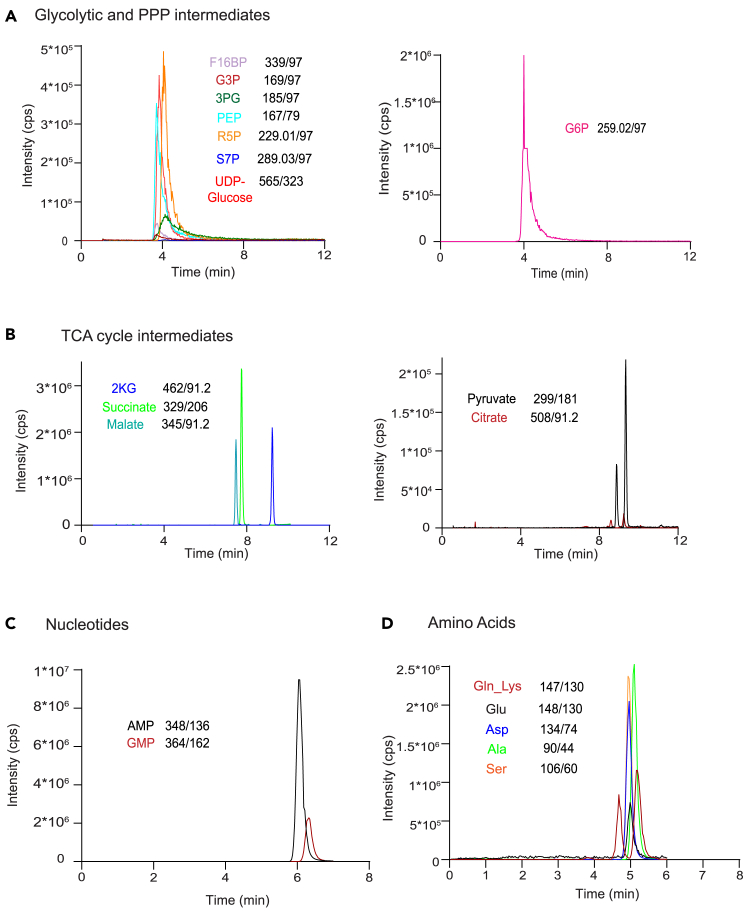
Representative chromatograms of select metabolites detected using LC/MS/MS metods Representative chromatograms for (A) glycolytic and PPP intermediates, (B) TCA cycle intermediates, (C) nucleotides and (D) amino acids measured using this protocol. The Q1/Q3 masses of each of these components, is also mentioned.

**CRITICAL:** Before starting, run few blanks (LC-MS grade water) to check for any background noise. Additionally, we also recommend injecting a suitable amount of a standard of a known concentration having all the metabolites being analyzed. Check for peak quality and signal intensities before proceeding with samples (see Figure 3 for representative chromatograms).

***Note:*** Glycolysis and PPP intermediates can be detected using the negative polarity mode.

23. Dissolve the samples in 1 mL of water. Inject a suitable amount for mass spectrometry analysis.
24. Separate the metabolites using the Synergi Fusion-RP column 80 Å (150 × 4.6 mm) on Shimadzu Nexera HPLC system using the following solvents: buffer A′, 5 mM ammonium acetate in H_2_O; and buffer B′, 100% acetonitrile.
25. Measure the steady-state and labeled metabolite amounts using the AB Sciex 5500 triple quadrupole mass spectrometer in MRM mode.

***Note:*** Amino acids and nucleotides can be detected using the positive polarity mode. The OBHA derivatized TCA cycle metabolites can be detected using positive polarity mode.

26. Dissolve the derivatized samples in 1 mL of 1:1 water: methanol. Dissolve the samples without derivatization in 1 mL of water. Inject a suitable amount for mass spectrometry analysis.
27. Separate the metabolites using the Synergi Fusion-RP column 80 Å (150 × 4.6 mm) on Shimadzu Nexera HPLC system using the following solvents: buffer A (aqueous phase), 99.9% H_2_O/0.1% formic acid; and buffer B (organic phase), 99.9% methanol/0.1% formic acid.
28. Measure the steady-state and labeled metabolite amounts using the AB Sciex 5500 triple quadrupole mass spectrometer in MRM mode.

### Data analysis

**Timing: ∼1 day**

This section describes parameters used for LC-MS/MS data analysis

29. Analyze the acquired data using MultiQuant software.
30. Make a quantitation method using data from the standard.
  a. Set retention times for the labeled metabolite based on that of the unlabeled counterparts.
  b. Set integration parameters as follows: Gaussian smooth width-2, RT half window-30 s, Minimum peak width-3 points, minimum peak height-0, noise percentage-75%, baseline sub. window- 2 min, report largest peak-yes.
31. Use this quantitation method, to analyze data from the samples.
32. Wherever needed, manual integration of peaks can be done.
33. Calculate the area under the curve for both labeled and unlabeled metabolites.
34. Calculate the percentage labeling as the ratio of the intensity of the specific labeled mass to the total intensity of all detected masses for the given metabolite.

## Expected outcomes

**Figure 4:**
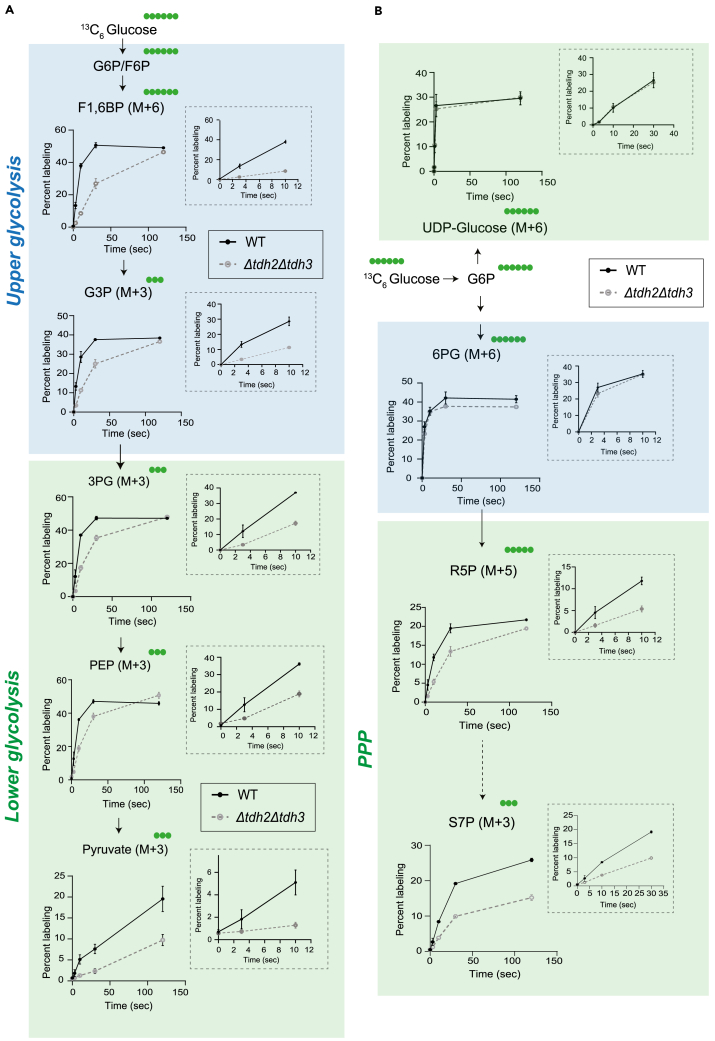
^13^C labeling kinetics in glycolysis and PPP (A) ^13^C label incorporation in glycolytic intermediates in WT and *tdh2Δtdh3Δ* (mutant with 50% reduced glycolytic flux) cells. WT and *tdh2Δtdh3Δ* cells were pulsed with ^13^C- glucose and metabolite extraction was carried out after indicated time intervals and ^13^C label incorporation in glycolytic intermediates was measured. Data represented as mean ± SD (*n* = 3). (B) ^13^C label incorporation in PPP and related metabolic pathways in WT and *tdh2Δtdh3Δ* cells. Data represented as mean ± SD (*n* = 3).

**Figure 5:**
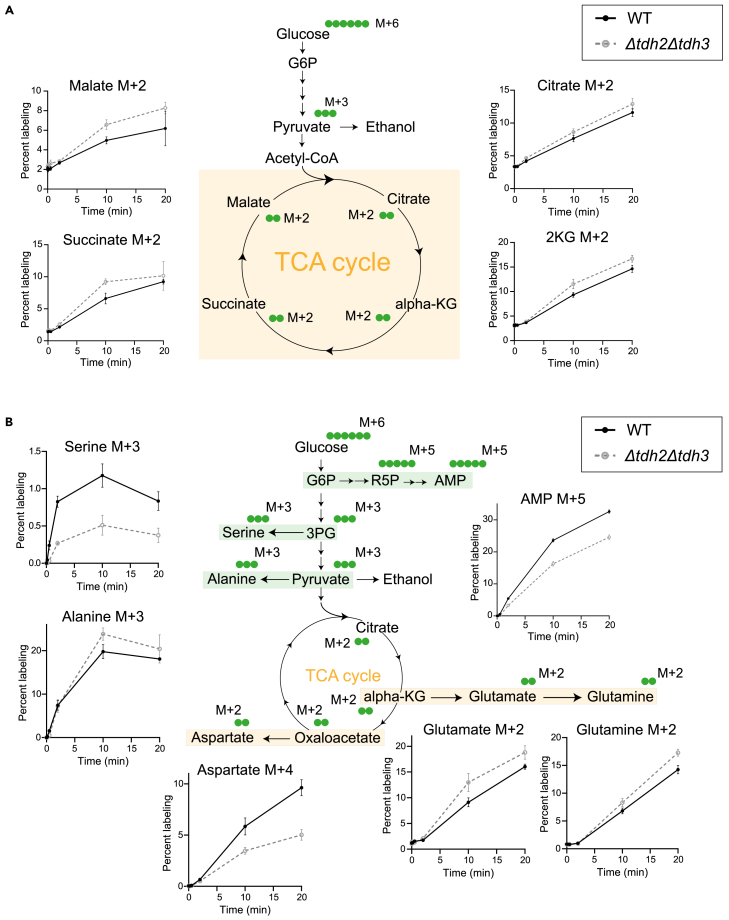
^13^C labeling kinetics in TCA cycle, amino acids, and nucleotides (A) ^13^C label incorporation in TCA cycle intermediates in WT and *tdh2Δtdh3Δ* cells. WT and *tdh2Δtdh3Δ* cells were pulsed with ^13^C-glucose and metabolite extraction was carried out after indicated time intervals and ^13^C label incorporation in TCA cycle intermediates was measured. Data represented as mean ± SD (*n* = 3). (B) ^13^C label incorporation in amino acids and nucleotides in WT and *tdh2Δtdh3Δ* cells. The metabolites are categorized and highlighted based on their biosynthetic origins. Data represented as mean ± SD (*n* = 3).

When performed correctly, the user will observe distinct temporal kinetics of label incorporation and saturation across glycolysis, the pentose phosphate pathway (PPP), the TCA cycle, amino acids, and nucleotides (Figures 4 and 5). This is because the kinetics of label incorporation and its saturation into newly formed metabolites depends on the kind of metabolic pathway involved. The organization (linear/cyclic), flux (high/low) and regulation could all influence this kinetics.^12^ In Crabtree positive yeasts, the rates of glycolysis can be ∼100 times that of the TCA cycle and operate at zero order kinetics. Hence, a short pulse with ^13^C labeled glucose would lead to immediate saturation in both glycolytic and PPP intermediates, while the kinetics would be much delayed into the TCA cycle, which operates at relatively slower rates along with cycling. Similarly, kinetics of label incorporation into distant outputs like amino acids and nucleotides will also be much slower.^12^ The differences in turnover rates and pathway dynamics will be clearly apparent, and can be quantified easily, especially as relative differences.

### Glycolysis

For the upper glycolysis intermediates (G6P, F6P, F16BP, G3P), labeling reached a steady state well under <10 s of adding ^13^C-glucose (Figure 4A). This rapid saturation means that extreme care must be taken by the users of this method, in order to accurately estimate flux changes in the upper glycolysis pathway. The use of stop-flow reaction chambers or similar high-resolution techniques can enable precise quantification of flux dynamics in these fast metabolic pathways especially in *in-vitro* cell lysates or purified enzymes. Implementing such approaches would significantly enhance the accuracy of measuring rapid label incorporation.

In contrast, the lower arm of glycolysis (when 3 carbon intermediates are formed) shows a linear increase in label incorporation up to 10 s after label addition (Figure 4B). This indicates that an 8–10 s time window is optimal for quenching metabolism and collecting samples to estimate flux through lower glycolysis. Using this approach, we can successfully detect and quantitatively assess differences in label incorporation between wild-type cells, and mutants with reduced glycolysis (in this case, yeast cells with two out of three GAPDH isoforms removed). Earlier studies suggested that a loss of these two isoforms results in a ∼50% decrease in glycolytic flux,^1^ which is more clearly corroborated in (Figure 4A).

Interestingly, pyruvate labeling continues to increase for several minutes after the tracer pulse. This indicates that the intermediates up to PEP turnover at very fast rates, while pyruvate synthesis also occurs at high rates, but with slower turnover. This is precisely as expected from the classical textbook models of glycolytic regulation,^14^ especially when the system is operating at near-saturation, indicative of ∼zero-order kinetics.

### Pentose phosphate pathway intermediates

For the oxidative PPP (oxPPP), label incorporation in the initial steps directly derived from glucose-6-phosphate (e.g., 6-phosphogluconate) reached a saturated steady state in under 10 s, similar to upper glycolysis (Figure 4B). Therefore, it is very difficult to practically estimate and quantify flux in these steps. However, downstream metabolites in the oxPPP exhibited a linear increase in labeling up to 10 s, making the 8–10 s window (to quench, and extract metabolites) very suitable for studying flux through the later steps of this pathway (Figure 4B).

### TCA cycle

In order for glucose-derived metabolites to enter the TCA cycle, pyruvate first forms, and enters the mitochondria and is converted to acetyl-CoA through the action of pyruvate dehydrogenase. Therefore, label incorporation from glucose to the TCA cycle will be expected to take longer than glycolytic rates. Using this method, we can observe that label incorporation in TCA cycle intermediates continued to increase (in a linear manner) for over 20 min following tracer addition, indicating relatively slower turnover/synthesis rates and flux through this cycle (Figure 5A). This reiterates that the TCA cycle operates at a much slower pace compared to glycolysis and oxPPP, and expectedly in a Crabtree positive cell, will not operate at saturation. It is also therefore relatively straightforward to estimate TCA cycle flux.

### Amino acids

Label incorporation into amino acids showed distinct patterns depending on their biosynthetic origins.^15^ For amino acids derived from glycolysis intermediates, such as alanine and serine, labeling increased linearly for the first 10 s and continued to increase more gradually over 20 min, reflecting rapid synthesis coupled with slower turnover (Figure 5B). In contrast, amino acids derived from TCA cycle intermediates, such as glutamate, glutamine, and aspartate, exhibited a steady increase in labeling over 20 min, indicating slower synthesis rates (Figure 5B).

### Nucleotides

Nucleotides showed a complex labeling pattern due to their biosynthetic origins derived from multiple precursors and pathways. Much of the carbon backbone of nucleotides comes from PPP intermediates (where labeling saturation is in ∼30 s, however, the nucleotide bases are synthesized using amino acid precursors such as glutamate and aspartate, and therefore the complete nucleotide synthesis will take a longer time). This can be observed experimentally, and the increase in label-incorporation into nucleotide monophosphates (e.g., AMP or GMP) follow labeling kinetics similar to the TCA cycle-derived amino acids, with a linear increase over 20 min (Figure 5B). This indicates slower nucleotide synthesis rates (compared to PPP flux), and the changes in label incorporation into nucleotide synthesis can be easily quantified, in order to estimate flux of nucleotide synthesis.

These findings reiterate the rapid turnover rates in pathways like glycolysis and oxPPP, compared to the slower dynamics in the TCA cycle, amino acid, and nucleotide synthesis. In this protocol, we clearly define time windows that can be used to estimate label incorporation (with ^13^C-glucose). With this, we provide a detailed and effective framework to quantitatively estimate and compare metabolic rates and fluxes for glucose-derived central carbon metabolic pathways. For the majority of the carbon/nitrogen pathways, our framework is now sufficient to set up a full flux experiment. The general time frames presented here cover these major classes and the time of experimental design.

This can easily be used to quantitatively estimate glucose metabolic flux for any Crabtree positive yeast and effectively be used to optimize or streamline applications dependent on fermentation rates.

## Limitations

While this method is extremely effective in estimating glycolytic flux in a single cell type, or comparing with one or two conditions, given the rapid time scales it is challenging to use this as a high throughput method, for large numbers of samples. Additionally, for absolute quantification of flux (of any of the intermediates or pathways), it is essential to run a range of concentrations of each metabolite standard, which can be challenging.

## Troubleshooting

### Problem 1

Errors between replicates.

### Potential solution

• Keep a timer for every time point and start it as soon as you add the ^13^C labeled glucose. Add the quenching buffer as soon as the timer stops.
• While resuspending the pellet after quenching and centrifugation, make sure to take all the suspension.
• Extraction buffer has ethanol which can stick to the walls of tips. Hence, aspirate carefully.

### Problem 2

Poor peaks for TCA cycle metabolites (step 17).

### Potential solution

TCA cycle metabolites are unstable in aqueous environments. Hence, we recommend derivatizing the metabolite extract (see step 18-22) before storing.

### Problem 3

High noise in blanks (step 23-28).

### Potential solution

Background noise could be due to contamination from the column. For this run few blanks or wash the column using 50% methanol (LC-MS grade) and check if the noise reduces. If not, the LC tubings the, ion source or the curtain plate may be contaminated.

### Problem 4

Multiple peaks in samples for a given metabolite with different retention times (step 24 and step 27).

### Potential solution

This usually indicates poor sample preparation and the peaks may be from a different compound. This can be avoided by taking care with sample preparation and clean up during metabolite extraction, and using a guard column in the HPLC.

### Problem 5

Peak broadening/tailing.

### Potential solution

This issue suggests that the LC column is in a poor condition. Regular cleaning of the column and storing it in adequate conditions can extend the usage period of the column.

## Resource availability

### Lead contact

Further information and requests for resources and reagents should be directed to and will be fulfilled by the lead contact: Sunil Laxman (sunil@instem.res.in).

### Technical contact

Technical questions on executing this protocol should be directed to and will be answered by the technical contact, Sreesa Sreedharan (sreesasreedharan95@gmail.com).

### Materials availability

This study did not generate new unique reagents.

### Data and code availability

Relevant data is available in Vengayil et al., 2024 https://doi.org/10.7554/eLife.90293.3.

## Acknowledgments

We acknowledge the extensive use of the NCBS, inStem, and CCAMP mass spectrometry facilities. Schematics were made using BioRender. S.L. acknowledges funding support from the DBT – Wellcome Trust India Alliance (IA/S/21/2/505922) and the S. Ramachandran National Bioscience Award for Career Development from the Department of Biotechnology, Government of India. S.S. acknowledges INSPIRE PhD fellowship support from the Science and Engineering Board (SERB), Department of Science and Technology, Government of India.

## Author contributions

Conceptualization, S.N., S.S., V.V., and S.L.; methodology, S.N., S.S., V.V., and S.L.; investigation, S.N. and S.S.; writing – original draft, S.N., S.S., and S.L.; writing – review and editing, S.N., S.S., and S.L..; funding acquisition, S.L.; resources, S.L.; supervision, S.L.

## Declaration of interests

The authors declare no competing interests.
